# Exploring the Impact of Foods and Dietary Patterns on Melanoma Risk

**DOI:** 10.7759/cureus.99404

**Published:** 2025-12-16

**Authors:** Matus Kalavsky, Shafiah Muna Abdul Gafoor

**Affiliations:** 1 Dermatology, West Hertfordshire Teaching Hospitals NHS Trust, Watford, GBR; 2 Dermatology, King's College Hospital NHS Foundation Trust, London, GBR

**Keywords:** diet and dermatology, malignant melanoma, primary prevention of skin cancer, ­skin cancer, skin cancer prevention

## Abstract

Malignant melanoma is the fifth most common cancer in the US. Ultraviolet (UV) radiation remains the primary environmental risk factor for melanoma; however, there is growing interest in exploring additional preventive strategies, particularly through diet. This narrative review aims to consolidate current evidence on the relationship between dietary patterns and melanoma risk, providing insights that may guide clinicians in offering dietary recommendations to their patients. Mediterranean and DASH (Dietary Approaches to Stop Hypertension) diets have been linked with potentially protective effects against melanoma. Similarly, the consumption of anti-inflammatory foods (fruits, vegetables, whole grains, legumes, nuts, herbs, spices, and other plant-based proteins) and regular coffee intake have shown associations with lower melanoma risk in observational studies. By contrast, alcohol consumption, adherence to a Western diet, and possibly high citrus fruit intake are linked to an increased risk of developing melanoma. While red and processed meats may be associated with a lower melanoma risk, their potential benefits are overshadowed by their associated risks for other cancers. The relationship between fish intake and melanoma risk appears to be complex, with some studies suggesting that canned tuna may be associated with an increased melanoma risk, whereas fried fish is linked to a potentially protective effect. Evidence remains inconclusive on the link between melanoma risk and nutrients such as selenium and vitamins A-E, as well as polyunsaturated fats and tea.

## Introduction and background

Malignant melanoma, a cancer originating from melanocytes (melanin-producing cells), predominantly affects the skin but can also develop in other organs such as the eyes and mucous membranes [1]. Its global incidence has been steadily increasing, representing a growing public health concern [2]. In the US, melanoma is the fifth most common cancer, with an average of 21.9 new cases per 100,000 men and women per year between 2018 and 2022, accounting for approximately 5% of all cancers [3]. During the same period, the death rate was two per 100,000 men and women per year [3].

The primary risk factors for melanoma include both genetic predispositions and environmental exposures, with ultraviolet (UV) radiation being the most significant environmental factor. Other environmental contributors include fair skin, sunbed use, and arsenic exposure [4]. In addition to these well-established factors, emerging evidence suggests that dietary patterns and specific nutrients may influence melanoma risk [5]. Dietary components may modulate melanoma development through mechanisms such as oxidative stress, inflammation, modulation of the host gut microbiome, and the effects of photosensitizing compounds, including psoralens found in certain fruits and vegetables. These biological pathways provide a scientific rationale for investigating diet as a modifiable factor in melanoma prevention.

Given the increasing interest in the potential role of nutrition in skin cancer prevention, this narrative review aims to synthesize current evidence on the relationship between dietary patterns, specific foods, and nutrients and melanoma risk. This review summarizes the current evidence on dietary factors and melanoma risk, highlighting potential associations that may guide future research and prevention strategies.

## Review

Literature search and inclusion criteria

To identify relevant literature for this narrative review, we searched PubMed and Google Scholar up to May 31, 2025, using a wide range of terms related to melanoma and diet. The list of keywords is as follows: "melanoma", "skin cancer", "diet", "nutrition", "dietary pattern", "Mediterranean diet", "Western diet", "DASH diet", "anti-inflammatory diet", "coffee", "tea", "alcohol", "fish", "meat", "red meat", "processed meat", "poultry", "citrus", "vitamins", "vitamin A", "retinol", "beta-carotene", "vitamin B", "niacin", "folate", "folic acid", "vitamin C", "vitamin D", "vitamin E", "nicotinamide", "PUFA", "omega-3", "omega-6", "fat", and "selenium". These terms were combined in various ways (e.g., “melanoma” and “coffee” or “skin cancer” and “vitamin D”).

Studies were included if they were peer-reviewed, published in English, involved human participants, and examined dietary or nutritional factors in relation to melanoma risk or incidence. Studies were excluded if they did not specifically report on melanoma-related outcomes, if they lacked sufficient information on dietary exposures, or were narrative reviews, editorials, or commentaries (except when highly relevant). Animal studies were also excluded.

Dietary patterns and melanoma risk

Diet is an important modifiable factor in cancer prevention, including melanoma. Dietary patterns, comprising various food combinations, can influence melanoma risk. For example, "Western" diets, characterized by high intakes of processed foods, refined grains, sugars, and unhealthy fats, are generally associated with increased cancer risk, while "traditional" or "healthy" diets, which emphasize fruits, vegetables, and fibre, are considered protective [6,7].

Mediterranean Diet

The Mediterranean diet, which includes abundant fruits, vegetables, whole grains, nuts, legumes, and olive oil, has been linked to a reduced melanoma risk. Similarly, anti-inflammatory foods, such as fruits, vegetables, whole grains, legumes, nuts, herbs, spices, and other plant-based proteins [8], may lower melanoma risk, possibly through modulation of the systemic immune response by natural phytochemicals and effects on the host gut microbiome [9]. Moderate dairy intake and limited red meat consumption further define this diet [10,11]. A large French study of 67,332 women demonstrated that greater adherence to the Mediterranean diet was associated with a significant reduction in melanoma risk [12]. Key components of this diet, such as olive oil and legumes, have shown possible protective effects against melanoma [13]. Moreover, a systematic review of observational studies found that higher intakes of fruits, vegetables, and fish were associated with a 34-46%, 40-57%, and 35-37% reduction in melanoma risk, respectively [14].

DASH Diet

Originally developed to reduce hypertension, the DASH diet (Dietary Approaches to Stop Hypertension) emphasizes fruits, vegetables, low-fat dairy, and fibre-rich foods [15]. A case-control study in Italy found a protective association between adherence to the DASH diet and reduced melanoma risk, particularly among women under 50 years old [13]. In addition, certain foods within this diet, such as legumes, olive oil, and eggs, were linked to reduced melanoma risk, while cereals, sweets, and chocolate were positively associated with an increased risk [13].

Western Diet

The Western diet, which is high in processed foods, refined grains, sugary drinks, and fried foods, has been associated with an increased risk of various cancers due to its high content of carbohydrates, sugars, and unhealthy fats [16]. Although research on the direct link between the Western diet and melanoma is limited, studies suggest a possible association between high consumption of sugars and refined carbohydrates and an increased melanoma risk. For example, an Italian study involving 45,148 participants showed that a diet rich in sugars and carbohydrates was linked to an increased melanoma risk [17]. In addition, two case-control studies found that increased consumption of cereals and sweets, particularly in women, was associated with a higher melanoma risk [13,18].

Micronutrients linked to melanoma risk

Antioxidants

Antioxidants are key in protecting melanocytes from oxidative stress caused by reactive oxygen species (ROS), which contribute to melanoma development [19]. Key antioxidants, including vitamins A, C, and E, selenium, and caffeine, have been studied for their potential roles in melanoma prevention.

Vitamin C and Citrus Fruits

Citrus fruits are rich in vitamin C and psoralens, which can act as photocarcinogens by intercalating DNA and causing mutations [20]. While vitamin C has shown some protective effects, the relationship with melanoma risk remains controversial. A U.S. study found that high dietary vitamin C intake from citrus fruits, particularly orange juice, was associated with an increased melanoma risk when consumed at higher doses [21]. Conversely, other studies, including an Italian case-control study, showed that vitamin C was linked to a decreased melanoma risk [22].

Vitamin D

Vitamin D, primarily synthesized via ultraviolet B radiation (UV-B) exposure, plays a role in regulating cell-cycle arrest, apoptosis, and DNA repair [23]. Despite these protective mechanisms, the role of vitamin D in melanoma prevention is debated. A U.S. randomized trial found mixed results; whilst high vitamin D intake was linked to an increased melanoma risk in men, elevated vitamin D consumption was associated with a decreased melanoma risk in women [24]. A systematic review found no significant association between vitamin D supplementation and melanoma risk [25]. However, some studies have suggested that small doses of vitamin D may offer protective effects [26].

Vitamins A and E

Retinol (vitamin A) and its precursors, carotenoids, have been linked to a reduced melanoma risk, particularly from dietary sources [27]. However, supplementation with vitamin A and beta-carotene has not shown consistent protective effects [28]. Vitamin E, with its antioxidative properties, has also been explored for melanoma prevention. However, most studies, including a case-control study, found no significant protective association, although food-based vitamin E intake showed some potential benefits in melanoma prevention [29,30].

Selenium

Selenium, found in various foods like fish, grains, and meat, has antioxidant properties that may protect against melanoma. However, studies on selenium and melanoma have produced mixed results. For instance, high selenium intake from tap water was associated with a greater melanoma incidence in a cohort study of Italian nurses [31]. By contrast, other studies found no significant link between selenium levels and melanoma risk [32]. These inconsistencies highlight the need for further investigation into selenium's role in melanoma risk.

Polyunsaturated Fats

The role of polyunsaturated fatty acids (PUFAs) in melanoma risk is complex. While omega-3 PUFAs, found in fatty fish, have been linked to reduced melanoma risk in some studies [33], omega-6 PUFAs were associated with increased risk of developing melanoma [34]. Interestingly, some studies indicate that diets high in both omega-3 and omega-6 fatty acids, such as the "meat, fish, and fat" dietary pattern, are associated with the development of thicker melanomas [35].

Nicotinamide

Nicotinamide, a form of vitamin B3, has shown promising results in reducing the risk of non-melanoma skin cancers, including basal cell carcinoma and squamous cell carcinoma [36]. Research suggests that nicotinamide works by enhancing DNA repair mechanisms and reducing inflammation in the skin, thereby preventing UV-induced damage [37]. While its protective effects against melanoma remain less clear, studies indicate that nicotinamide may offer some level of protection against non-melanoma skin cancers, potentially due to its ability to modulate immune responses and promote skin cell repair. However, further research is needed to determine its role in melanoma prevention specifically.

Beverages and protein sources

Coffee 

Coffee has been shown to offer potential chemopreventive effects against several cancers, including colorectal, breast, and endometrial cancers, although results remain inconsistent [38]. The protective properties of coffee are primarily attributed to polyphenolic antioxidants and anti-inflammatory compounds, such as caffeine, cafestol, kahweol, and chlorogenic acids [39]. A study in Norway (n = 50,757) found that coffee consumption demonstrated an association with a significant reduction in melanoma risk in women but not in men [40]. Furthermore, moderate intake of filtered coffee (one to three cups/day) was associated with a lower melanoma risk [41]. High caffeine intake has also been linked to a reduced melanoma risk among healthcare workers [42]. A large European prospective study (n = 500,000) demonstrated a strong inverse association between coffee consumption and melanoma risk in men [43], a finding corroborated by a multi-ethnic cohort study in Hawaii and Los Angeles [44]. Supporting these results, systematic reviews and meta-analyses suggest a potential protective effect of caffeinated coffee against melanoma [45,46].

Tea

The relationship between tea consumption and melanoma remains unclear. Large-scale studies (n = 35,369 and n > 500,000) found no significant association between tea intake and melanoma risk [43,47]. However, a smaller case-control study (n = 609) indicated that daily tea consumption might reduce melanoma risk [33]. These mixed findings warrant further investigation into tea's role in melanoma prevention.

Alcohol

Alcohol is a known carcinogen that accounts for approximately 4% of global cancer cases and increases the risk of various cancers, including melanoma [48]. A U.S. case-control study (n = 1,067) found that high alcohol consumption was linked to an increased melanoma risk [49]. Specifically, higher intake of white wine and liquor was associated with a greater melanoma risk in postmenopausal women (n = 59,575), particularly with consumption of seven or more drinks per week [50]. A pooled analysis of 210,252 participants also demonstrated an elevated melanoma risk with alcohol intake [51]. Similarly, a meta-analysis of 14 case-control and two cohort studies indicated that any alcohol consumption increased melanoma risk compared to abstinence [52], with a Mendelian randomization study further suggesting a causal relationship between alcohol intake and melanoma [53]. Alcohol may elevate melanoma risk through its metabolite, acetaldehyde, which promotes photosensitization of the skin [54].

Fish

The role of fish consumption in melanoma risk is complex. A systematic review found that higher fish intake was associated with a 35-37% reduced risk of melanoma [14]. However, a large U.S. prospective study (n = 492,186) reported a positive association between fish intake and melanoma risk, particularly with canned tuna consumption [55]. More recently, a U.S. study found that higher fish intake was linked to increased risks of malignant melanoma and melanoma in situ, with specific positive associations for tuna and non-fried fish, while fried fish appeared to be associated with a reduced melanoma risk [56].

Meat

Consumption of red meat (pork, beef, lamb, veal, and mutton) and processed meat (which is treated to improve flavour or shelf life through methods such as salting, curing, or smoking) has been linked to an increased risk of several cancers, including those of the breast, colorectum, lung, and liver [57]. Surprisingly, two U.S. prospective studies found an inverse association between red and processed meat consumption and melanoma risk, with higher intake associated with reduced melanoma risk [58]. Similarly, a large prospective study (n = 500,000) observed a negative association for processed meat, although no significant link was found for red meat consumption specifically [59]. This potential protective effect could be due to retinol found in red meat, which is known to inhibit tumour growth by limiting cell proliferation and inducing apoptosis [60,61]. However, smaller case-control studies from the U.S. and Italy found no significant association between red or processed meat consumption and melanoma [62,63]. Given these mixed findings, it is difficult to conclude a strong relationship between red or processed meat consumption and melanoma, and any potential protective effect may be outweighed by the established risks of these meats in other cancers [64].

## Conclusions

The relationship between diet and melanoma risk is complex and not yet fully understood. The existing studies have demonstrated associations of the Mediterranean diet, DASH diet, anti-inflammatory foods, and regular coffee consumption with lower melanoma risk. By contrast, alcohol consumption, adherence to a Western diet, and possibly high citrus fruit intake have been associated with increased risk of melanoma. Although red and processed meat consumption may be associated with lower melanoma risk, their potential advantages are outweighed by the increased risk they pose for other cancers. The relationship between fish consumption and melanoma risk appears to be complex. In a large prospective cohort study, higher intake of canned tuna and non-fried fish was linked to an increased melanoma risk, whereas fried fish was associated with a potentially protective effect. However, these findings must be interpreted cautiously due to potential confounding factors that were not accounted for. These include individual sun-related behaviours, such as sunscreen or sunbed use; individual risk factors, such as mole count or a childhood history of severe sunburn; as well as potential contaminants in fish, such as arsenic or mercury.

Moreover, dietary epidemiological studies are subject to inherent limitations, including recall bias, measurement error in food frequency questionnaires, and residual confounding from unmeasured variables. These challenges may partly explain the mixed results observed for many micronutrients and food groups. Therefore, more well-designed prospective studies and systematic reviews that control for these biases are necessary to clarify the protective and risk-enhancing roles of dietary factors in melanoma prevention. Until more conclusive evidence is available, nutritional counselling should continue to promote general healthy eating habits, including adequate intake of fruits, vegetables, and fibre, while advising limiting alcohol consumption, particularly in individuals with other established risk factors for melanoma.
